# Ecological and cosmological coexistence thinking in a hypervariable environment: causal models of economic success and failure among farmers, foragers, and fishermen of southwestern Madagascar

**DOI:** 10.3389/fpsyg.2015.01533

**Published:** 2015-10-13

**Authors:** Bram Tucker, Tsiazonera, Jaovola Tombo, Patricia Hajasoa, Charlotte Nagnisaha

**Affiliations:** ^1^Department of Anthropology, University of GeorgiaAthens, GA, USA; ^2^Department of History, Université de ToliaraToliara, Madagascar; ^3^Department of Geography, Université de ToliaraToliara, Madagascar

**Keywords:** culture, risk, causal cognition, unpredictability, traditional knowledge, cosmology, Madagascar

## Abstract

A fact of life for farmers, hunter-gatherers, and fishermen in the rural parts of the world are that crops fail, wild resources become scarce, and winds discourage fishing. In this article we approach subsistence risk from the perspective of “coexistence thinking,” the simultaneous application of natural and supernatural causal models to explain subsistence success and failure. In southwestern Madagascar, the ecological world is characterized by extreme variability and unpredictability, and the cosmological world is characterized by anxiety about supernatural dangers. Ecological and cosmological causes seem to point to different risk minimizing strategies: to avoid losses from drought, flood, or heavy winds, one should diversify activities and be flexible; but to avoid losses caused by disrespected spirits one should narrow one’s range of behaviors to follow the code of taboos and offerings. We address this paradox by investigating whether southwestern Malagasy understand natural and supernatural causes as occupying separate, contradictory explanatory systems (target dependence), whether they make no categorical distinction between natural and supernatural forces and combine them within a single explanatory system (synthetic thinking), or whether they have separate natural and supernatural categories of causes that are integrated into one explanatory system so that supernatural forces drive natural forces (integrative thinking). Results from three field studies suggest that (a) informants explain why crops, prey, and market activities succeed or fail with reference to natural causal forces like rainfall and pests, (b) they explain why individual persons experience success or failure primarily with supernatural factors like God and ancestors, and (c) they understand supernatural forces as driving natural forces, so that ecology and cosmology represent distinct sets of causes within a single explanatory framework. We expect that future cross-cultural analyses may find that this form of “integrative thinking” is common in unpredictable environments and is a cognitive strategy that accompanies economic diversification.

## Introduction

The subsistence farmer, forager, and fisherman contemplating choice of crops, livestock, and prey inevitably faces the reality that crops fail, livestock sicken and die, foragers and fishermen come home empty handed, and selling prices in the marketplace drop. Sometimes the causes of economic failures are easily observable. Crops may fail because of drought or pests or because the farmer did not spend enough time weeding, and a fisherman may return to shore with low catch due to unfavorable winds. In other cases the reasons for failure may be less apparent. A farmer may lose a bountiful crop the night before she intends to harvest due to a sudden windstorm or grasshopper swarm. A fisher may unexpectedly find that a batch of fish prepared for smoking have turned rotten.

Human minds in their social contexts search for patterns and meaning behind the causes of success and failure. People search for covariations between environmental cues and subsistence outcomes, in order to better predict, and thus seek to control, their harvests of crops and wild resources. When unexpected failure happens, people ask deeper questions such as why *my* field was destroyed and not my neighbor’s. Covariation theories (Nisbett and Ross, 1980, p. 90–112), knowledge of the base rate frequencies with which things happen (Cheng, 1997; Griffiths and Tenenbaum, 2005), and learned mechanisms for causality (Ahn et al., 1995) are the building blocks composing cultural models of causality (Waldmann et al., 2006) that people use to make important subsistence choices and understand their fortunes.

Subsistence risk is exactly the type of domain where one would expect what Legare et al. (2012) call “coexistence thinking,” the simultaneous application of natural and supernatural models of causality to explain why things happen. The ethnographic record is replete with examples of farmers, hunter-gatherers, and fishermen using a mix of ecological knowledge and cosmological knowledge when making important decisions (Rappaport, 1968; Poggie and Pollnac, 1988; Lansing, 1991; Malinowski, 1992[1948]; Dove, 1993; Birkes et al., 2000; Orlove et al., 2000; Hunn et al., 2003). Contrary to Victorian and modernist notions that supernatural causality constitutes primitive thought (Tylor, 1958[1871]) or childish thought (Piaget, 1928) that is eventually replaced by a more sophisticated understanding of the clockwork of the natural world, a recent review of experimental studies demonstrates that coexistence thinking is pervasive in modern, urban, educated contexts, and that adults often endorse supernatural causes *more frequently* than children (Legare et al., 2012).

How and when one may explain fortune and failure with wind and rain versus angels and ancestors is an open question, one that we pursue in this article. In a recent review by Legare et al. (2012) they present a typology of coexistence thinking. In “target-dependent thinking,” reasoners pick and choose natural and supernatural causes to explain different components of a phenomena or in different contexts. For example, in a study of AIDS etiology in South Africa, Legare and Gelman (2008) quoted one informant as saying that “witchcraft may cause a disease that looks like AIDS.” In the context of people’s understanding of the origins and diversity of life, a reasoner may justify divine creation for humans because of a belief in the eternal soul but endorse natural selection for a purportedly soulless animal (Evans et al., 2011). Target dependence suggests that natural and supernatural causal systems are separate and sometimes in conflict with one another.

In “synthetic thinking,” natural and supernatural causes are alike and co-exist within a single causal system, so that unsafe sex and witches both cause AIDS, and natural selection and God both influence life’s diversity. Synthetic thinking implies that natural and supernatural forces are unified into a single explanatory system. Ethnographic descriptions of some indigenous South American cosmologies suggest that some people understand persons, animals, and spirits to be commensurate anthropomorphic agents (Descola, 1996; Viveiros de Castro, 2000; but see Ramos, 2012), thus eschewing the natural/supernatural dichotomy.

“Integrative thinking” refers to causal models where supernatural forces drive natural forces; witchcraft causes people to have unsafe sex and get AIDS, and God created the diversity of life by means of natural selection. The best-known ethnographic example of integrative thinking is probably Evans-Pritchard’s (1937) description of Zande witchcraft in southwestern Sudan. Zande understand that the cause of a granary collapsing is that termites have eaten the wooden supports. But the ultimate cause, the reason why termites attacked *that* particular granary so that it fell when someone happened to be resting in its shadow, is witchcraft, an evil power unleashed by a neighbor’s secret, jealous thoughts. In integrative thinking, natural and supernatural causes are categorically differentiated but occupy the same explanatory system.

Although a population may apply all three modes of coexistence thinking to the same causal problem, in the studies reviewed by Legare et al. (2012, p. 790) subjects do not apply each mode with the same frequency. For example integrative explanations are the least common in studies of people’s understanding of death as biological cessation versus continuity into an afterlife (Harris and Giménez, 2005; Astuti and Harris, 2008), presumably because biological death and ancestral continuity are contradictory (although Astuti and Harris insist that Vezo fishermen have no problem accepting both).

This demands the question whether the frequency with which people use target-dependent, synthetic, or integrative thinking is the result of differences in the causal problems being solved versus differences in the populations, or both. Variability across causal problems is likely to be the results of the ecology of risk associated with each problem. The causal problems involved in rural subsistence strategies, such as forecasting rainfall, harvests, or fishing returns, are influenced by ecological factors such as climatic variability, predictability and the visibility of cues and covariations foreshadowing future events. Inter-population variability would likely be due to different culturally learned causal models that compose what anthropologists have called traditional ecological knowledge (Dove, 1993; Birkes et al., 2000; Orlove et al., 2000; Hunn et al., 2003) and cosmology (Descola, 1996; Viveiros de Castro, 2000; Howell, 2012).

The case study from southwestern Madagascar presented in this paper is intriguing because characteristics of the ecology and culture seem to favor contradictory strategies to minimize risk. The ecology (specifically, the climate) is characterized by extremely high variability and unpredictability, so that the best way to avoid risk is diversification and behavioral flexibility. The culture (specifically, the cosmology) explains risk as caused by disgruntled spiritual agents, so that people live in a state of spiritual insecurity, “the sense of danger, doubt, and fear arising from an awareness of exposure to invisible forces acting to cause misfortune” (Ashforth, 2010, p. 43). The best way to avoid risk associated with spiritual insecurity is to restrict the range of one’s behaviors.

Antarctic winds, cyclones, and el Niño events conspire to give Madagascar one of the most variable and unpredictable climates in the world (Wright, 1999), what Dewar and Richard (2007) call “a hypervariable environment.” In a comparison of monthly rainfall data, Dewar and Richard (2007) found that rainfall is less predictable across 15 Malagasy weather stations contrasted to 15 stations in continental Africa, where predictability was evaluated after Colwell (1974) as variability that does not covary with month or season (low contingency). Toliara, in southwestern Madagascar near where our study is set, had the second lowest predictability score with a Colwell’s *P* of 0.281. This means that even with exceptional ecological knowledge a subsistence decision-maker can only ever have 28% confidence in monthly rainfall forecasts. Farmers, foragers, and fishermen throughout the world often cope with ecological unpredictability by diversifying their portfolios of crops, prey, and market activities (Winterhalder et al., 1999), and this is a common strategy in southwestern Madagascar (Tucker, 2001, 2006, 2007a,b; Tucker et al., 2010, 2013).

Ancestors, spirits, and a distant creator God observe the living and reward and punish people according to how well their behavior demonstrates respect. Crops fail and foragers and fishers come home empty handed because people transgress the behavioral code of taboos, offerings, other “cosmo-rules” (to borrow a phrase from Howell, 2012), either mindfully or accidentally.

A southwestern Malagasy person who wishes to avoid misfortunes caused by disrespected spiritual agents should observe dietary taboos, follow the astrological calendar of good and bad days for subsistence labor, respect elders and traditional magico-religious specialists, and not travel too far from the houses of the protective spiritual agents, in tombs, enchanted trees and rocks, and in the miniature houses that spirit mediums construct behind their homes. But reducing the risks caused by extreme weather conditions requires tracking a changing environment and planning for multiple contingencies. It may require exploiting a range of resources including those that are taboo; scheduling labor flexibly, even when the astrological calendar indicates that the day is not propitious for working; and traveling far from one’s homeland to exploit distant fields and patches and traffic goods to market, distant from the terrain of protecting spirits.

In this paper we consider the question of how southwestern Malagasy people combine causal models involving wind and rain which seem to advise flexibility with causal models involving God and spirits that seem to prescribe conformity. Legare et al.’s (2012) modes of coexistence thinking suggest three possibilities. One possibility is that our informants use target-dependent thinking; they carry contradictory models around in their heads simultaneously without justifying one with the other. As they apply each model in different circumstances they bounce between conforming and flexible strategies. A second possibility is that they consider rain, wind, God, and spirits to belong to a single category of causes, composing a synthetic causal model with a consistent internal logic. A synthetic model could prescribe both conformity and flexibility in different circumstances, for example, providing ritual ways to permit diversification, for example, to excuse exploitation of taboo resources or permit distant travel. A third, integrative possibility is that natural and supernatural forces belong to separate categories but a single causal model, where supernatural forces drive natural outcomes. Using an integrative model, farmers, foragers, and fishers may make subsistence choices using ecological knowledge, but then apply cosmological knowledge after the fact to explain the successes and failures of their forecasts and strategies. Our previous ethnographic observations suggest that all three possibilities are plausible.

We examine these possibilities in light of the findings from three field studies conducted in southwestern Madagascar. In the first study we asked groups of informants to rate and explain the risk associated with different crops, prey, and market activities. We predicted that the groups would rate risks concordantly with each other, indicating a shared body of cultural knowledge. A second prediction, following Malinowski (1992[1948]), was that people would cite more supernatural causes for riskier activities. The first prediction is confirmed for the sample at large with the exception of Vezo coastal fishers. In contrast to our second prediction, informants listed almost entirely natural causes of risk such as rainfall and pests.

In the second study we asked individuals to explain the reasons why a hypothetical man in a vignette harvested more than his friend. We conducted an economic and a religious version of the vignette to see whether subjects would provide more natural causes in the former and more supernatural causes in the latter, consistent with target-dependent thinking. Instead, in both versions of the vignette informants provided primarily supernatural causes like God and ancestors. When taken together with the findings of Study 1, this suggests that our informants understand activities as responding to natural causes while personal successes and failures result from supernatural forces, consistent with integrative thinking.

The third study used a card sorting activity to examine the causal flow of natural and supernatural factors, including God, ancestors, weather, and harvest (similar to Lynch and Medin, 2006). A target-dependent thinker with competing causal epistemologies might depict independent natural and supernatural chains of causes. A synthetic thinker who treats natural and supernatural factors as coinfluential would likely depict bilateral causal interactions among natural and supernatural factors. Instead, results support a hierarchy of causal factors with supernatural forces driving natural forces (integrative thinking). The specific form of the causal flow involves humans begging ancestors to beg God for good weather resulting in a good harvest.

We propose that when reasoning about activity risk divorced of personal context southwestern Malagasy reason within a natural causal submodel or level represented by the last two links in this chain (weather → harvest), but when reasoning about people they employ the whole chain, (humans → ancestors → God → weather → harvest). Thus in southwestern Madagascar, natural and supernatural forces are categorically distinct but occupy a single causal model rather than forming competing epistemologies. We conclude with a discussion of the significance of these findings for understanding the influences of culture and ecology on coexistence thinking, and by considering whether “natural” and “supernatural” mean the same thing in rural Madagascar as they seem to mean in Western scholarly discourse.

Research into the effects of culture and ecology on how people understand the causal influence of natural and supernatural factors on economic outcomes is significant for several reasons. Decision-making under risk is a classic topic within economics where it is modeled using probability theory (Bernstein, 1996), despite a growing body of evidence that people do not think in terms of probability distributions (Kahneman and Tversky, 1979; Gigerenzer and Selten, 2001). This article offers a cognitive perspective on subsistence risk, and explores how culture and ecology co-influence people’s understanding of risk. More generally, it is significant to understand how human minds in their social contexts understand and organize causal knowledge because this forms shared concepts of reality (ontology) and ways for understanding this reality (epistemology). Studies of the cultural and ecological determinants of coexistence thinking may help us address the classic question whether all humans basically understand the world as working in the same general way (psychic unity of humankind) or whether thought is an infinitely variable cultural construction (cultural relativism; Stocking, 1987; see discussion in Bender and Beller, 2011; Bender et al., 2012).

## The Cultural Context

### Southwestern Malagasy

The people of Madagascar are unified by a single language and a similar set of traditional cosmological beliefs and practices, yet there is significant inter-regional variation in dialects, customs, habits, beliefs, norms, and social institutions. This article pertains specifically to Malagasy people living in the arid southwest between the provincial capital of Toliara and the port of Morombe. We refer to these people collectively as “southwestern Malagasy,” although they refer to themselves as Masikoro, Mikea, and Vezo. These are not ethnic groups in a traditional sense, for “ethnic” implies that identity is inherited from parents and is perceived to be intrinsic and essential, whereas identity in southwestern Madagascar is more flexible than this (Astuti, 1995, 2001; Astuti et al., 2004).

Southwestern Malagasy commonly claim that to be a Masikoro means that one is a farmer, a herder, and savanna dweller, while a Mikea is a forest-based hunter-gatherer, and a Vezo is a coastal gatherer, fisher, and sailor. Mikea informants have often told us that “Mikea” is a livelihood (*velomampò*) and not a “kind of person” (*karazan’olo*) and that all hunter-gatherers in the world are Mikea. Astuti (1995) reports similar statements from Vezo about fishing and fishermen.

In practice this simple classification meets with perpetual exceptions. There are savanna farmers who call themselves Mikea, coastal Vezo who farm, and Masikoro fishermen. Households also tend to be diversified, with different members practicing a range of farming, foraging, fishing, herding, and marketing activities, with activity portfolios changing over time. We have argued that most of the contradictions are resolved if we recognize that people also cite family, clan, and community histories as reasons for claiming Masikoro, Mikea, or Vezo identity. These histories trace back to the precolonial Andrevola kingdom. People identify as Masikoro in part because their ancestors were vassals to the Andrevola kings, while Mikea and Vezo recall ancestors who resisted royal domination by hiding in the forest or sailing away to sea (Yount et al., 2001; Tucker, 2003). In this article we assume that Masikoro, Mikea, and Vezo constitute a single cultural group where members move in and out of multiple subsistence options.

### Risk and Ecology

Southwestern Madagascar is a semi-arid limestone shelf bordered by the Onilahy and Mangoky Rivers. The landscape is diverse. Within the span of a 100 km east–west transect one may traverse grassy savanna and savanna woodlands; the dense, dry, deciduous Mikea Forest (*Alamikea*); the lakebeds, dunes, and thorn forests of the Namonte Basin; followed by coastal mudflats, mangroves, the shallow Bay of Fagnemotse; then white sandy beaches and a barrier reef. Farmers plant crops like maize (*Zea mays*), manioc (*Manihot esculenta*), and rice (*Oryza sativa*) in rainfed and irrigated savanna fields, in forest swiddens, and in gardens in the Namonte lakebeds on the coast. Foragers dig wild *ovy* tubers (*Dioscorea acuminata*) in the deciduous forest, gather estivating tenrecs (African hedgehogs, *Echinops telfairi*), and fish in the fresh waters of the Namonte Basin and lake Ihotre. On the coast people gather mud crabs (*Scylla serrata*) in the mangroves, collect octopus (*Octopus cyanea*) and sea cucumbers (*Holothuria* and *Scabra* sp.) in the shallows before the reef, and fish for finfish, shark, and sea turtles with lines and nets. Masikoro and Mikea sell an average of 45% of their production in local markets and to exporters, while Vezo sell an average of 87% (Tucker et al., 2010, 2013).

Our previous research into subsistence risk in the region finds that agriculture produces more food by quantity but at considerably greater risk than foraging or fishing (Tucker et al., 2010). A useful way to illustrate this is with a thought experiment. Imagine that an individual will spend 90 days on just one subsistence activity. 90 days of labor spent cultivating maize would result in a two-hectare field that would produce an average of 1862 kg maize, or roughly 6.8 M calories. By contrast, 90 days spent digging wild ovy tubers yields an average of 864 kg or 1.0 M calories. However, the maize farmer invests all her hopes on a single harvest, one that is highly dependent on good rainfall and sparse pests. The wild tuber forager harvests every day, distributing her risk over 90 foraging trips. If a tuber forager has a string of bad days she can move to a part of the forest that received more rain or switch prey, options that are not available to the farmer. In a set of simulations following this logic, we found that agriculture tends to be an order of magnitude more risky than foraging and fishing (Tucker et al., 2010). When asked to rate the risk of their subsistence activities, Masikoro, Mikea, and Vezo in 24 focus groups generally agreed that agriculture is riskier than foraging and fishing (Tucker et al., 2013).

### Previous Evidence for Covariation Theories

In a previous study we tested whether southwestern Malagasy have consistent covariation theories linking rainfall and the outcomes of farming, foraging, and fishing activities using an historical matrix exercise in fourteen communities (10 Mikea, two Vezo-Mikea, and two Masikoro) in 1999 (Tucker, 2007a). The method involved creating a tabular grid of playing cards on the ground in which rows represent the past 5 years, the first column represents rainfall, and subsequent columns represent crops and prey. A group of informants was instructed to work together to pour sand on to each card representing the quantity rainfall or harvest in each year. Working as a group engendered conversation. Of 95 comments that we recorded, 22 were statements of quantity (e.g., “a lot”), 28 were statements of events (“the year of the big cyclone”), and 45 were statements of rules (“when there is a lot of rain, there is no honey”). Rule statements and rank-order correlations between the sand piles in the rainfall and harvest columns were quite consistent. Groups agreed that, “when there is no rain, there is no maize,” “manioc hates rain,” “rain destroys rice irrigation schemes,” and “fish drink water, too” (meaning, there are more fish in rainy years). This evidence suggests that southwestern Malagasy have shared ecological knowledge of the causal interactions between rainfall and economic outcomes.

### Cosmology

In the traditional cosmology of southwestern Madagascar the creator God *Ndragnahare* (called *Zanahary* or *Andriamanitra* elsewhere in Madagascar) is distant and people interact most commonly with ancestors (*raza*) and spirits that possess mediums in trance ceremonies (*doane*). These invisible forces observe the personal lives of human beings and their judgment results in triumphs or failures.

Recent theories about the evolution of religion have proposed that rewards and punishments by omniscient “high moralizing gods” (Norenzayan, 2013) or by a “broad spectrum” of moralizing supernatural agents (as Watts et al., 2015 argue is more appropriate to Austronesian cultures) function to reinforce cooperative norms by rewarding niceness and punishing selfishness. Malagasy cosmology fits imperfectly within this scheme. In Madagascar, the ancestors and other spirits are not omniscient, they may deceive and be deceived, and the moral code they reinforce is primarily one of respect, for God, for the universe, and especially, for the spiritual agents themselves and their earthly representatives, elders, spirit mediums, and diviners (Ruud, 1960; Feeley-Harnik, 1991; Sharp, 1993; Graeber, 2007; Astuti and Bloch, 2015).

For example, a man may commit a selfish act like killing his neighbor’s livestock out of jealously. Ancestors and other spirits are unlikely to punish this offense. The livestock owner may consult a diviner who may, through divination with grains (*sikily*), learn from the spirits that the neighbor is guilty and that the neighbor has “bad ideas” (*raty hevitse*) or “a bad soul” (*raty say*). Still, spirits issue no punishment. But had the bad person killed cattle that had been allocated for sacrifice to the ancestors, this would have been a clear offense that the ancestors would have punished forthwith.

Although ancestors, spirits, and God rarely directly reward interpersonal niceness and punish meanness, the code of respect that they enforce is prosocial, for it creates the traditional social structure. Ancestors may be capricious and selfish but they are essentially good because they connect the living to tombs (*lolo*), to the land (*tanindraza*), to one’s clan (*firazagna*), and one’s community (*filongoa*), securing one’s social identity, right to material and social resources, and membership in a larger intergenerational corporate program (Bloch, 1971, 2008; Feeley-Harnik, 1991; Graeber, 2007). Participating in clan activities and maintaining community sentiment requires interpersonal niceness (and sometimes meanness to enemies or criminals), even though interpersonal acts are not the major concern of supernatural agents^1^.

People consult with the spirit world by performing clan ceremonies and livestock sacrifice to honor ancestors under the direction of the clan head (*mpitokazomanga*), through trance ceremonies with mediums (*tromba*), through divination under the guidance of the diviner (*ambiasa*, called *ombiasy* elsewhere in Madagascar), and, for a rare few, through knowledge of astrology (*andro, vinta*). People demonstrate respect for the supernatural primarily by respecting a code of dangers or taboos called *faly* (*fady* elsewhere in Madagascar) that limits dietary choices, sexual behavior, clothing, mobility, permissible speech, and other behaviors (Ruud, 1960). Each individual has her own personal set of *faly* associated with place, clan, astrological destiny (*vinta*), and magical charms (*aoly*). People acknowledge a common calendar of good and bad days for different activities (*andro*). Most people know that one should refrain from work on Mondays and Thursdays; some also know the taboos associated with particular month-day combinations, and the correspondence of dates with stellar positions.

In a survey of 550 Masikoro, Mikea, and Vezo in the study region we found that 17% claimed to be Lutheran (mostly Masikoro) and 17% claimed to be Catholic (mostly Vezo). While some Christians publicly eschew traditional religion, many self-identifying Christians in the region host and attend traditional ceremonies, consult with clan heads, spirit mediums, and diviners, and show every other sign of conforming to traditional Malagasy cosmological expectations.

## Study 1: Popular Concepts of Risk for Subsistence Activities

The first study has been published elsewhere framed in pursuit of a different set of questions (Tucker et al., 2013); the study is reviewed here in light of its contributions to the topic of coexistence thinking. Study 1 explored shared concepts of risk for different crops, prey, and market activities, and the causes for activity risk. In the context of group interviews we asked people to define risk, list their subsistence activities, provide reasons why each activity is risky, and then rate the risk of each activity on a four-point scale. If our informants use coexistence thinking when reasoning about subsistence risk then the groups should cite a combination of natural and supernatural causes for activity risk. A second prediction, inspired by Malinowski (1992[1948]), was that people would cite more supernatural causes for higher risk activities. Our third prediction was that if informants have a consistent body of knowledge about risk, groups should rate risks concordantly.

### Sample

We interviewed groups rather than individuals because we were interested in shared, public knowledge, and group interview settings encourage individuals to provide “normal” answers (Smithson, 2000). We convened 24 sex-segregated focus groups in 12 villages in 2008. The villages were a mix of Masikoro farmers (*N* = 3 sites), Mikea forager-farmers (*N* = 6 sites), Vezo coastal fishers (*N* = 2 sites), and Tandroy farmers (*N* = 1 site; Tandroy migrated to the region from southern Madagascar in the 1930s). Group interviews occurred after a meeting with the townspeople (*fokon’olo*) in which we explained our research objectives and sought community consent. We divided the pool of willing adults into male and female groups of 6–10 each and sought oral consent to conduct the research. Two Malagasy researchers of the same sex as the informants posed the questions.

First we asked people to define risk. Two words for risk had come to our attention in previous research: *risike*, from the French *risque*, and *kitahitahy*, which literally means “little blessing” but could also mean potential fortune. We did not seek to distinguish one term from the other but simply asked the informant to explain *risike* or *kitahitahy* and let them choose which word to use^2^. Then we asked the assembly to list their most significant subsistence activities and to rate each subsistence activity on a four-point scale: not risky (*tsy misy risike*), low risk (*risidrisike avao*), risky (*misy risike*), or very risky (*risike mare*). As the groups discussed their ratings we asked them to provide reasons (causes) for why each activity is potentially risky. Research participants were served cookies and coffee, and no other compensation was provided. This method was approved by the Institutional Review Board at the University of Georgia (2007-10358-0).

### Results

From the 24 focus groups we received 31 definitions of risk, which fall roughly into four categories: risk is something you must face in order to gain something (*N* = 19), risk means you might win or you might not (*N* = 12), risk is something that requires courage to face (*N* = 12), and risk is what happens when many factors predict an outcome (*N* = 4). Of the 31 definitions, only three, belonging this final category, mentioned supernatural causes.

The groups listed 53 unique crops, prey, and other economic activities. Average activity risk ratings are calculated over different *N*s because some activities were listed by only one group while others were listed by all 14. **Table 1** summarizes the risk ratings for activities listed by four or more groups. The seven agricultural activities are all listed among the top 12 riskiest activities alongside collecting sea cucumbers and marine line fishing and big game hunting (bushpig, shark). Across all 53 activities the average ratings were, for agriculture, *M* = 1.96, *SD* = 0.80; for forest foraging, *M* = 0.98, *SD* = 1.05; for marine foraging, *M* = 1.94, *SD* = 0.91, and for marketing activities, *M* = 1.09, *SD* = 1.12. For those activities listed by at least four groups, the ratings were, for agriculture, *M* = 2.09, *SD* = 0.69; for forest foraging, *M* = 1.03, *SD* = 1.05; for marine foraging, *M* = 2.00, *SD* = 0.80, and for marketing activities, *M* = 1.13, *SD* = 1.07.

**Table 1:** A rank ordered summary of groups’ average risk ratings for activities listed by four or more groups.

Activity	Crop/prey scientific name	Subsistence mode	*N* (focus groups who rated)	Average risk (0 = not risky–3 = very risky)	Standard deviation (disagreement)
Lesser hedgehog tencec	*Echinops telfairi*	Forest foraging	7	0.14	0.38
Trapping birds	Various *sp.*	Forest foraging	4	0.25	0.50
Wage labor replanting rice		Market	7	0.43	0.79
Digging *ovy* tubers	*Dioscorea acuminata*	Forest foraging	8	0.50	0.76
Freshwater fishing, line	*Tilapia* sp.	Forest foraging	4	0.75	0.96
Gathering and selling fuelwood		Market	9	0.78	0.97
Wage labor, rice tilling		Market	6	0.83	0.98
Hunting mouse lemur	*Microcebus murinus*	Forest foraging	4	1.00	0.82
Tobacco retailing		Market	4	1.00	0.82
Coffee vending		Market	9	1.56	1.01
Marine finfish, with net	Various sp.	Marine fishing	6	1.67	0.81
Shop keeping		Market	11	1.73	1.19
Honey gathering	*Apis mellifera*	Forest foraging	8	1.75	0.89
Sea cucumber gathering, night	*Holothuria* sp.; *Scabra* sp.	Marine fishing	4	1.75	1.26
Pumpkin	*Cucurbita pepo*	Agriculture	6	1.83	0.41
Sea cucumber gathering, day	*Holothuria* sp.; *Scabra* sp.	Marine fishing	6	1.83	0.98
Gathering octopus	*Octopus cyanea*	Agriculture	7	1.86	0.38
Vohem beans	*Phaseolus* sp.	Agriculture	7	1.86	0.38
Sweet potato	*Ipomoea batatas*	Agriculture	9	1.89	0.60
Onion	*Allium cepa*	Agriculture	5	2.00	0.71
Maize	*Zea mays*	Agriculture	12	2.08	0.90
Marine finfish, line	Various sp.	Marine fishing	5	2.20	0.45
Manioc	*Manihot esculenta*	Agriculture	14	2.21	0.70
Rice	*Oryza sativa*	Agriculture	9	2.56	0.73
Bushpig hunting	*Potamocorus larvatus*	Forest foraging	5	2.80	0.45
Shark netting	Unidentified sp.	Marine fishing	4	3.00	0.00

*Adapted from Tucker et al. (2013, p. 401–402).*

**Table 2** summarizes inter-group agreement as measured by Cohen’s Kappa. This analysis was conduced on a dataset of all unique combinations of paired ratings for each product (*N* = 606). On average there was a 42.74% agreement across all pairs of ratings, which is significantly greater than the 29.72% agreement expected by chance κ = 0.18(606), *p* ≤ 0.001. Landis and Koch (1977, p. 165) label this “moderate” consensus. Inter-group agreement was moderate for men κ = 0.32(135), *p* ≤ 0.001, just above traditional 0.05 alpha levels for women κ = 0.08(139), *p* = 0.066, and significant for Masikoro *k* = 0.22(48), *p* = 0.005, Mikea κ = 0.16(168), *p* ≤ 0.001 and Tandroy κ = 0.80(9), *p* ≤ 0.001, but not Vezo κ = 0.10(43), *p* = 0.153.

**Table 2:** Summary of analysis of agreement between all pairs of risk ratings for the same activity, using Cohen’s Kappa.

Sample	*N* (pairs rating activity)	Agreement (%)	Agreement expected by chance (%)	Kappa	*z*	*p*
All	606	42.74	29.72	0.18	7.70	0.000
Women	139	30.94	25.52	0.08	1.50	0.066
Men	135	58.52	39.03	0.32	5.14	0.000
Masikoro	48	45.83	30.73	0.22	2.59	0.005
Mikea	168	38.10	26.53	0.16	3.48	0.000
Vezo	43	44.19	37.75	0.10	1.02	0.153
Tandroy	9	88.89	44.44	0.80	3.34	0.000

*Adapted from Tucker et al. (2013, p. 402).*

In total, informants offered 239 causes for risk. Nearly all were natural causes, including pests (*N* = 79), rainfall (*N* = 35), dangerous encounters with animals, bandits, or gendarmes (*N* = 26), and risk of injury (*N* = 18). Only two of the 239 causes were supernatural. A Mikea woman said success fishing for snakehead fish depends on the astrological significance of the day (*andro*). Another Mikea woman said that honey foraging is risky because one might encounter a *tsiboko*, an undead creature with glowing red eyes.

### Discussion

Our first prediction was that groups would supply a mix of natural and supernatural causes for activity risk, consistent with coexistence thinking. Much to our surprise, both risk definitions and causes of risk were almost entirely earthly and secular. This could suggest that our informants preferentially rely on ecological knowledge when reasoning about activities, or that the domain of subsistence is immune from coexistence thinking. It is also possible that the public nature of group methods discouraged discussion of supernatural causes. Our second prediction, that southwestern Malagasy would provide more supernatural causes for riskier activities, is not supported given that almost no supernatural causes were provided.

Our third prediction, that our informants would largely agree about the riskiness of their activities, received partial support, for there was moderate agreement in risk ratings across the whole sample, excluding Vezo. The low agreement in the Vezo sample is likely due to the fact that Vezo are the most specialized on one mode of subsistence, marine foraging, and listed exclusively fishing and market activities. The ordered list of perceived risk in **Table 1** shows that activities roughly cluster by mode, so that forest foraging activities are lowest risk, marine fishing is moderately risky, and agriculture is the most risky. Southwestern Malagasy may agree more on the order of risk among these modes rather than among individual activities within these modes. We do not have a satisfactory explanation for why there was less agreement among the female focus groups than the male groups. We would expect men and women to have similar experience with most of these crops and prey, for men and women work in the same fields and forage for the same terrestrial resources (excluding bushpig, which are exclusively hunted by men), although the division of labor is clearer for marine fishing, where men exploit deeper waters.

## Study 2: Differential Economic Success Vignette

The purpose of the next study is to get a clearer picture of when and how southwestern Malagasy employ coexistence thinking. While study 1 asked about *activity* risk, in this study we asked individuals to discuss why *persons* succeed or fail. The method involved a vignette about two men, Reolo and Tsiato^3^, who are good friends but not close kin, who discover one day that Reolo consistently harvests more than Tsiato. Informants were asked to provide possible causes for Reolo’s greater success. We conducted two versions of the vignette, one framed around a market scene and the other around a funeral. If the domain of subsistence is immune to coexistence thinking, then we would predict that informants would provide primarily natural causes, as they did in the previous study. Target dependence would be demonstrated if our informants primarily provided ecological causes when the vignette was framed around a market scene and supernatural causes when framed around a funeral scene. Synthetic thinking should lead to informants providing a mix of natural and supernatural causes as if these were commensurate kinds of influencing factors. If participants favor supernatural causes (in contrast to Study 1), then a few interpretations are possible. They may be using a kind of target-dependent thinking with which they explain activity risk (Study 1) and personal risk (Study 2) with competing causal models. Or it could be that they understand harvests to be the result of natural forces where natural forces are ultimately influenced by supernatural forces that reward and punish individuals (integrative thinking).

### Sample

These data were collected in the Mikea community of Bevondrorano and the Vezo village of Lamboara in 2014. Bevondro is a series of Masikoro and Mikea villages clustered around two small lakes on the eastern edge of the Mikea Forest, where people divide their time among forest foraging, lake fishing, and floodplain cultivation of manioc, sweet potatoes, and rice. Bevondrorano is one of the Bevondro villages, settled within the past 2 years by Mikea displaced by the new Mikea Forest National Park^4^. Bevondrorano is home to Mikea we have known since the 1990s from forest camps and the villages of the Namonte Basin. The Vezo village of Lamboara, located on a small island in the mouth of the Bay of Fagnemotse, is among the older settlements on the Vezo coast, having been established in the 19th century.

The samples for Studies 2 and 3 are described in **Table 3**. For Study 2 we recruited 12 adult participants in Bevondrorano (nine women, three men) and 24 in Lamboara (14 women, 10 men). Nine Lamboara people also participated in Study 3. Due to time constraints we did not attempt random sampling. These samples represent roughly a quarter of the total population in both communities.

**Table 3:** Sample characteristics for Studies 2 and 3.

Village study	Sample size		Age^1^			Mean Frequency of church attendance^2^	Mean Years of formal education	Wealth^3^	
					


	Total	Women	Young adult	Adult	Old adult			Low	High
Bevondrorano (Mikea)									
Study 2	12	9	4	7	1	0.4	1.2	10	2
Lamboara (Vezo)									
Study 2	24	14	3	17	4	1.3	4.3	16	8
Study 3	22	10	2	15	5	1.4	4.0	10	12

*^1^Based on a visual estimate by the authors.*

*^2^Average of responses scaled as 0 = never, 1 = sometimes, 2 = frequently.*

*^3^Informants asked if they own eight common assets (land, cattle, oxcart, ocean-going canoe, shark fishing net, radio, generator, and telephone). Low = owns 0–3 assets; high = owns 4–6 assets. Mean = 1.8 assets, max = 6 assets.*

### Method

We interviewed individuals in the shade of our camp or near their homes. We attempted to talk to people in private, but participants were sometimes accompanied by a spouse or friend, and by young children in their care. After obtaining oral consent we began with a series of questions about the individual’s household size, education, frequency of church attendance, and ownership of a set of productive and luxury assets, from which we calculated a wealth score^5^. The instrument is presented in its original Malagasy form and in English in supplemental materials.

We administered an economic and a religious version of our vignette, each to half the sample. The script for the economic version of the vignette is as follows.

I am going to tell you an imaginary story about two men, Reolo and Tsiato. Reolo and Tsiato are good friends, but they come from different families, and they live in different villages that are rather far away from each other. What both men have in common is that they both work constantly to manage their household economy, particularly by frequent buying and selling in marketplaces. In fact, it is at the market that the two men often see each other, and share news over a cup of coffee.
One day when they talk together about their lives, they are surprised to learn that Reolo consistently harvests more crops than does Tsiato, whether it is maize, rice, or manioc. Tsiato harvests much less than his good friend does.

The second version of the vignette was religiously framed.

I am going to tell you an imaginary story about two men, Reolo and Tsiato. Reolo and Tsiato are good friends, but they come from different families, and they live in different villages that are rather far away from each other. What both men have in common is that they both work constantly to manage family affairs, particularly by attending many of family ceremonies, such as circumcision, rites of filiation, and funerals. They often see each other in the ceremonies, where they exchange news.
One day when they talk together about their lives, they are surprised to learn that Reolo consistently harvests more crops than does Tsiato, whether it is maize, rice, or manioc. Tsiato harvests much less than his good friend does.

With Vezo research subjects, we substituted harvest of “maize, rice, manioc” with harvest of “fish, octopus, and sea cucumbers.”

It is important that Reolo and Tsiato do not live close to one another so that their economic activities are not influenced by the same weather. It is important that they are not kin so that they do not share the same ancestors. The vignettes were accompanied by drawings of either two Malagasy men shaking hands in a marketplace or at a funeral.

After telling the story we first asked the informant to voluntarily *list* reasons why Reolo experienced better success than Tsiato (because participants may have different explanations for gains versus losses, we consistently emphasized Reolo’s success relative to Tsiato’s failure). Then we presented a list of possible causal factors and asked the informant whether she would *endorse* each cause. We provided seven natural factors (e.g., rainfall, wind, and pests), three social factors (age, poverty, jealous neighbors), and six supernatural factors (e.g., God, ancestors, taboo transgression, etc). These factors are listed, along with a summary of results, in **Table 4**. When the exercise was completed informants received a small cash gift (1000 MGA, $0.50 USD, equivalent to 10 cups of coffee). This method was approved by the University of Georgia’s Institutional Review Board (MOD00001573).

**Table 4:** Frequency that different natural and supernatural factors were listed or endorsed by Mikea and Vezo informants.

	Factors listed voluntarily by informants		Factors endorsed by informants when listed by the researcher		Factors listed and endorsed	
			
	Mikea	Vezo	Mikea	Vezo	Mikea	Vezo
*N*	12	24	12	24	12	24
**Natural factors**						
Rainfall	3	0	3	3	6	3
Hard work	5	5	3	9	8	14
Pests^M^/Wind^V^	0	0	6	3	6	3
Good land^M^/good canoe^V^	2	0	1	3	3	3
Weeds^M^/good nets^V^	0	0	1	4	1	4
Inherited land^M^/good swimmer^V^	1	0	2	1	3	1
Fertilizer^M^	0	0	0	0	0	0
**Social factors**						
Age	0	0	2	4	2	4
Poverty	0	0	2	4	2	4
Jealous neighbors	0	0	2	2	2	2
Didn’t do bad things to others^∗^	0	1	0	0	0	1
Good parents^∗^	1	2	0	0	1	2
**Supernatural factors**						
Ancestors	7	20	2	3	9	23
Possessing spirits	2	1	7	7	9	8
God	11	23	1	1	12	24
Magic	5	4	5	4	10	8
Other people’s magical attack	0	0	1	0	1	0
Transgression of taboos	0	0	8	8	8	8
Astrological destiny	3	0	5	17	8	17
Church attendance	0	0	10	9	10	9
“Anjara” (turn)^∗^	0	10	0	0	0	10
Astrological day^∗^	0	1	0	0	0	1

*Each informant listed 0–5 causes; when presented with the remaining causes in this list, each endorsed 2–14 additional causes. M, asked to Mikea only; V, asked to Vezo only. ^∗^Factors introduced by informants that were not part of our original list*.

### Results

Informants voluntarily listed 0–5 causes (*M* = 3.0, *SD* = 1.2), and endorsed an additional 2–14 causes (*M* = 7.0, *SD* = 2.7). Because of this variation in total number of causes listed and endorsed, all analyses that follow examine the percent of supernatural causes out of the total causes the individual listed or endorsed.

There were no significant differences by version of the vignette in the percent of causes that were supernatural, listed [economic version *M* = 82.9, *SD* = 28.4; religious version *M* = 75.4, *SD* = 28.5*; t* = 0.790(36), *p* = 0.435] or endorsed [economic version *M* = 76.6, *SD* = 22.1; religious version *M* = 72.8, *SD* = 18.4; *t* = -1.035(36), *p* = 0.308]. We combine results from both versions in the remaining analyses.

There were also no statistical differences in the responses of women versus men, listed [women *M* = 80.4, *SD* = 25.9; men *M* = 76.5, *SD* = 33.2; *t* = 0.384(36), *p* = 0.703] or endorsed [women *M* = 76.2, *SD* = 20.3; men *M = 71.8*, *SD* = 20.0; *t* = -1.018(36), *p* = 0.316]; by wealth, listed [low *M* = 77.1, *SD* = 31.4; high *M* = 83.8, *SD* = 18.9; *t* = -0.632(36), *p* = 0.531] or endorsed [low *M* = 72.8, *SD* = 21.0; high *M* = 79.3, *SD* = 17.5; *t* = 1.740(36), *p* = 0.091]; by frequency of attendance to a Christian church, listed [never *M* = 79.0, *SD* = 19.6; sometimes *M* = 80.1, *SD* = 29.9; always *M* = 76.0, *SD* = 34.3; *F* = 0.06(*N* = 36, df = 2), *p* = 0.944] or endorsed [never *M* = 77.2, *SD* = 17.9; sometimes *M* = 74.1, *SD* = 21.7; always *M* = 73.4, *SD* = 19.8; *F* = 0.14(*N* = 36, df = 2), *p* = 0.874]; or by whether participants also participated in Study 3 [listed, no *M* = 75.4, *SD* = 31.5, yes *M* = 88.3, *SD* = 15.3, *t* = -1.235(36), *p* = 0.224; endorsed, no *M* = 72.3, *SD* = 20.9, yes *M* = 80.7, *SD* = 17.1, *t* = 0.721(36), *p* = 0.476].

Participants listed several causes that we did not anticipate: it was Reolo’s *turn* to succeed (*anjara*, an idiom for luck related to astrology, *N* = 10), Reolo did not do bad things to neighbors *N* = 1, Reolo had good parents *N* = 1, and Reolo had a favorable astrological day *N* = 1 (we had asked about astrological destiny, *vinta*, but neglected to ask about the related concept of *andro*, a propitious day).

Participants primarily listed supernatural causes for Reolo’s superior success: God *N* = 34 and ancestors *N* = 27, followed by an important earthly cause, working hard *N* = 10. Only three Mikea listed rainfall, and no one listed wind, pests, weeds, or fertilizer, although they were endorsed fairly frequently when listed by the researcher. Mikea listed a lesser percentage of supernatural causes than did Vezo [Vezo *M* = 85.4, *SD* = 25.1; Mikea *M* = 66.1, *SD* = 31.0; *t* = 2.01(36), *p* = 0.052], although they endorsed a similar percentage of supernatural causes [Vezo *M* = 76.9, *SD* = 20.3; Mikea *M* = 70.0, *SD* = 19.5; *t* = -0.980(36), *p* = 0.334].

### Discussion

When asked why *individuals* succeed or fail, our informants primarily provided supernatural causes, in contrast to Study 1. Because informants cited natural and supernatural causes at similar frequencies regardless of the version of the story it does not appear that people apply competing causal models to economic versus religious problems (target-dependent thinking).

One could argue that the economic and religious versions are not really all that different because, while they present different contexts for Reolo and Tsiato’s meeting (market, ceremony), they are both about economic outcomes (harvest). It is interesting, then, that informants responded to them primarily with supernatural causes; had one of the vignettes featured a supernatural outcome (e.g., angry, dishonored ancestors) rather than a harvest, it seems most likely this would also be explained with supernatural causes.

The contrast between Study 1 and Study 2 suggests either that southwestern Malagasy apply competing knowledges to activity risk and personal risk (target dependence) or that they understand activities to respond in predictable ways to natural causes while the natural causes themselves are influenced by supernatural rewards and sanctions for the farmer, forager, or fisherman (integrative thinking). Study 3 attempts to distinguish between these possibilities.

## Study 3: Sorting Causes into a Flow

This study used a sorting activity to discern people’s concepts of the causal flow linking natural and supernatural forces. Vezo informants chose cards to represent God, ancestors, weather, and harvest (catch of fish). They were asked how pairs of cards influence one another, and were then asked to sort the cards into a causal flow. The method is similar to one used by Lynch and Medin (2006) to examine how U.S. undergraduates, nurses, and energy healers understand the physical and psychosocial causes of heart attack and depression, and our predictions follow their example. If southwestern Malagasy apply competing knowledges to activity risk and personal risk (target dependence), then we would expect them to create two separate chains, one with God and ancestors influencing harvest and the other with weather influencing harvest. If our informants understand no real existential difference in natural and supernatural forces (synthetic thinking), we would expect them to trace multiple bilateral interactions among natural and supernatural forces, and alternate their effects within chains. Integrative thinkers would present a hierarchy of causes with supernatural forces driving natural forces; the cards would form a single chain indicating that while supernatural and natural forces are different, they occupy the same causal system.

### Sample

The sample included 22 Vezo adults (9 women, 13 men) in the village of Lamboara. As with study 2, recruitment was non-random and covered approximately a quarter of the adult population in the village. Nine subjects were also participants in study 2, which they may have done before or after this study. Descriptive statistics are in **Table 3**.

### Method

As with study 2, we attempted to interview individuals privately but at times they were accompanied by a spouse, friend, or children. After obtaining oral consent from the interviewee we presented her with with six colored cards (white, black, red, yellow, blue, green) and quizzed her on the names of the colors (one man could not name the colors; the interview was terminated and he received the cash gift). We then asked the informant to choose one card to represent God (*Ndragnahare*), one to represent ancestors (*raza)*, one to represent weather (*toets’andro*), and one to represent harvest of fish (*vokatse fia*). The informant was quizzed to see if she remembered what each color represented.

Then we presented each of the 12 pairwise combinations of two cards to the research participant and asked whether one force could influence the other. “Influence” was difficult to translate into the local dialect of Malagasy. We used the verb *mikomandy* (from the French verb *commander*; the French verb implies less force than the English “command,” with a meaning closer to “request”), *mandily* (a synonym for *mikomandy*), or *magnina* (meaning to matter, to be the reason for). Informants switched among these terms, suggesting that their meanings are roughly equivalent in this context.

Next we asked the informant to place the four cards in rank order by power. Once this was completed we asked them how all four cards influenced one another together. We ended the exercise by adding an additional card, usually the yellow one, with the announcement that this represents spirits that possess people (*doane*). The subject was then instructed to add this card to the causal flow. When the exercise was completed each informant received a small cash gift (1000 MGA). This method was approved by the University of Georgia’s Institutional Review Board (MOD00001573).

### Results and Discussion

The majority of the 22 participants chose to represent God with the white card (*N* = 17), ancestors and weather with black or red (ancestors six black, five red; weather eight black, five red), and harvest with blue (*N* = 9) or green (*N* = 6). Informants frequently substituted “wind” (*taiky*) for weather, consistent with the importance of wind in the marine economy. As **Table 5** presents, informants were unanimous that God may influence ancestors and weather but not vice versa, and most said that God may influence harvest (*N* = 20) but not vice-versa (*N* = 22). There was disagreement as to whether ancestors influence weather (*N* = 3) or weather influences ancestors (*N* = 4), but the majority answered no to both questions. In the process of discussing the pairwise interactions, 14 of 22 volunteered that ancestors cannot influence weather directly, but must beg God to change the weather. An additional three people offered that God cannot influence harvest directly, but can only command the weather that in turn influences harvest.

**Table 5:** Results of 12 questions asking, does factor X influence factor Y? Does Y influence X? The table reports frequencies of yes responses.

First ->	God	Ancestors	Weather	Harvest
God		0/22	0/22	0/22
Ancestors	22/22		4/20 ^∗^	1/22
Weather	22/22	3/20^∗^		0/22
Harvest	20/22	22/22	22/22	

*^∗^These combinations were accidentally omitted in two cases.*

Twenty one informants sorted the cards by relative power, with much agreement. All 21 placed God as the most powerful force; 20 positioned harvest as the least powerful; and 20 ranked weather and ancestors as intermediate in power (13 favored ancestors, 6 favored weather, and 2 insisted they were of equal power).

**FIGURE 1:**
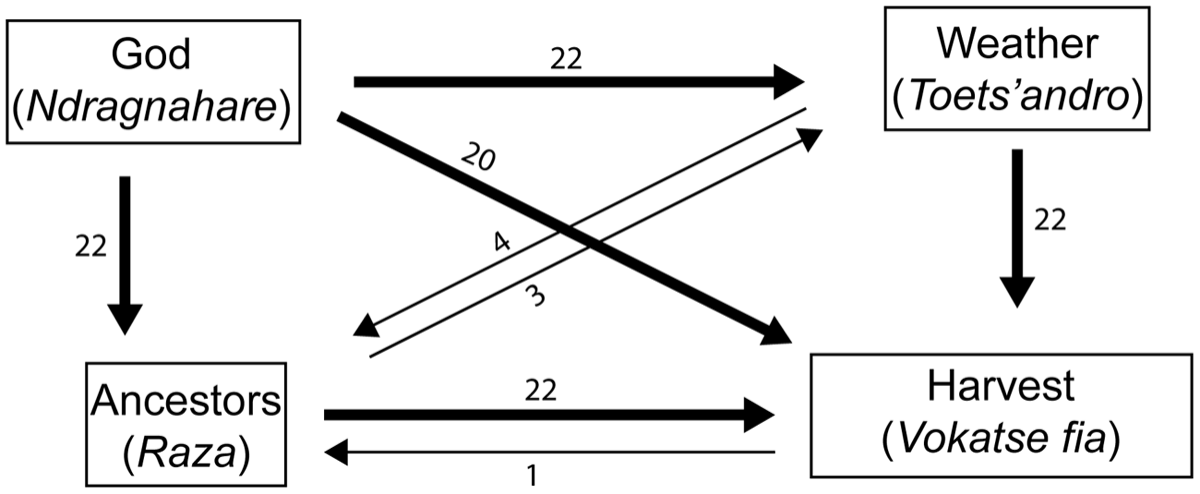
**The causal flow among God, ancestors, weather, and harvest implied by informant’s responses to pairwise questions, does X *influence* Y? See text for the Malagasy meaning of “influence**.”

**FIGURE 2:**
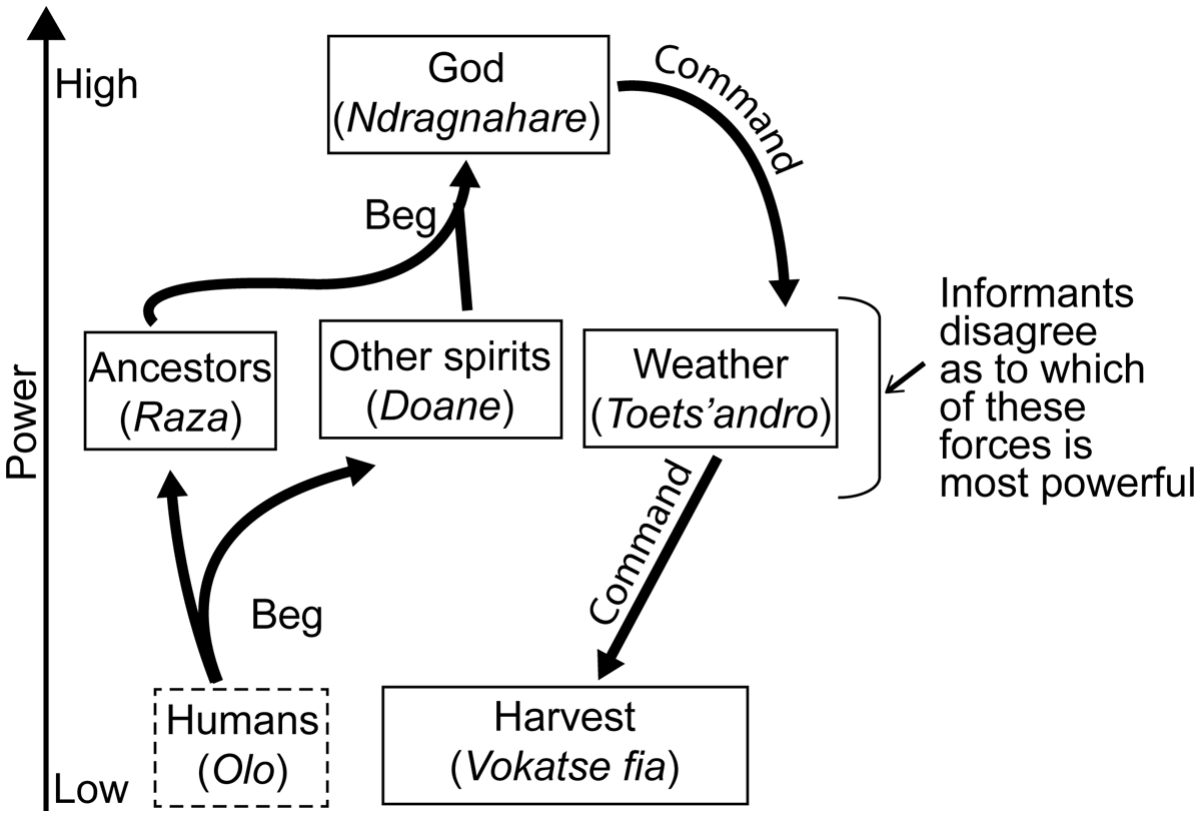
**When asked specifically to organize the cards to a causal flow, 11 informants produced this pattern**.

From the pairwise interactions in **Table 5** we may infer the causal flow represented in **Figure 1**, in which God influences everything, God is influenced by nothing, and God, weather, and ancestors command the harvest of fish. When presented with all four cards and asked to arrange cards into a causal flow, the majority of research participants who completed the exercise produced the different looking diagram in **Figure 2**. The method was a challenging task, both because we had not worked out a clear procedure for instructing informants in this exercise^6^, and because some informants did not consider themselves experts on such topics. Fourteen people completed the task. The arrangement in **Figure 2** was generated independently by 11 individuals.

There are two major differences between the causal flows depicted in **Figures 1** and **2**. First, in the second diagram ancestors (and possessing spirits) may influence God. This key difference is explained by the verb our informants used to explain how ancestors influence God: by begging (*mangatake*), which is less forceful than commanding (*mikomandy, mandily*) and causing (*magnina*). Although we did not include a card to represent people, participants also indicated that people beg ancestors and spirits to beg God on their behalf, so that God may command the weather to influence the harvest. The second contrast is that in **Figure 2** there are no direct arrows linking ancestors and God to harvest. In **Figure 2** ancestors and God *do* influence harvest, consistent with **Figure 1**, but via the chain of ancestors → God → weather → harvest of fish. This is also consistent with the comments made by several informants that God cannot influence harvest directly but may only do by influencing the weather, and ancestors cannot influence the harvest directly, but must ask God to change the weather.

The causal flow represented in **Figures 1** and **2** suggests that natural and supernatural forces are arranged in a hierarchy, and that supernatural forces drive natural forces, consistent with integrative thinking.

## General Discussion

The studies reviewed here support a specific form of integrative thinking in which people ascribe activity risk to natural factors (Study 1) and personal risk to supernatural factors (Study 2), and supernatural and natural forces form a hierarchy within a single explanatory system (Study 3). Natural and supernatural causal forces occupy distinct categories, in contrast to recent descriptions of indigenous American thinking (Descola, 1996; Viveiros de Castro, 2000), but do not form competing epistemologies, as seems to be the case for many Westerners when explaining life as resulting from evolution versus divine creation (Evans et al., 2011), and death as biological cessation versus afterlife (Harris and Giménez, 2005; Astuti and Harris, 2008).

We argue that when southwestern Malagasy reason about the risk of farming, foraging, or fishing activities, they reason within a natural submodel or level of explanation represented by the last two links in **Figure 2**’s causal chain, weather → harvest. In an environment where rainfall is only predictable 28% of the time, someone with good ecological knowledge may successfully forecast weather and economic outcomes one third of the time, while someone with bad knowledge leaves her fate completely to chance. In the rural economy there is often a long delay between cause (rainfall) and effect (crop yield, prey abundance) so that causality is difficult to observe. Instead, people learn natural causal models from others through cultural transmission (Boyd and Richerson, 1988). That supernatural and natural forces are categorically distinct, and the application of natural factors to activity risk, allows people to share information about drought tolerance and prey behavior while minimizing the distortions of personal and social concerns.

But even with really good ecological knowledge, the highly unpredictable climate means that people’s predictions will often fail. By ascribing *personal* successes and failures with the whole causal chain (humans → ancestors → God → weather → harvest), the Malagasy person may make sense of successes and failures without doubting the validity of the ecological knowledge. We predict that integrative thinking may be a common cognitive strategy accompanying economic diversification in risky environments.

This argument is consistent with our ethnographic observations. In our previous research in the region we have had countless discussions with farmers, foragers, and fishermen about their subsistence decisions and economic strategies, including such questions as when should one plant swidden maize, how many grains to plant, what to do if the crop does not germinate, how many times a manioc field should be weeded, how long manioc should be left in the ground to mature, what variety of rice is most pest-resistant, how to find wild *ovy* tuber patches, how to tell when *ovy* patches have been exhaustively harvest, what size gillnets are best for different fish species, etc. Almost unanimously, informants responded with the sorts of agronomic and ecological factors that an outsider without knowledge of local cosmology would expect. Meanwhile, our field notes describing the social life of Mikea, Masikoro, and Vezo communities are replete with accounts of people worrying about ancestors, navigating taboos, seeking benedictions in ceremonies, and sorcery accusations.

Southwestern Malagasy seek creative ways to diversify their income sources while also piously following their cosmo-rules. We provide two examples. First, the *doane* spirits that possess mediums typically cannot tolerate the presence of chickens, so spirit mediums have a taboo forbidding them to eat or raise chickens (*faly akoho*). When a spirit medium provides charms and other prescriptions to a client, the medium commonly insists that the client also avoid contact with chickens, lest the potency of charms be annulled. Chickens and eggs have become profitable market commodities, due to the ease with which they are produced and stable prices. Clients of spirit possession commonly request that the *doane* spirit allow a waiver of the chicken taboo, as long as chickens are only raised to convert to cash. A second example involves labor migration among Vezo fishers, many of whom, during the past decade, have undergone the long, dangerous ocean voyage to the region of Maintirano, half the island’s length to the north, where, according to the rumors, fisheries offer inexhaustible plenty and vendors buy ocean products at good prices. The separation from family tombs, elders, and possessing spirits adds significant risk on top of the physical risks of a long ocean voyage to an uncertain opportunity. To balance pious conformity to cosmo-rules with behavioral flexibility, Vezo mariners consult with one or more magico-religious leaders, including spirit mediums, diviners, and clan heads, to request special dispensation from ritual duties and protective charms.

The results reported here suggest that there may be some interesting variation within our sample, among farmers, foragers, and fishermen. Vezo fishing communities had less consistent evaluations of risk than Masikoro, Mikea, or Tandroy (although, as we argued, this could be because Vezo only listed fishing and marketing activities and there may be greater differences in risk among modes than activities). Mikea listed more natural causes for Reolo’s superior success than did Vezo. Masikoro, Mikea, and Vezo, and farmers, foragers, and fishermen, may approach the world somewhat differently for both ecological and cultural reasons. Agriculture, foraging, and fishing represent different intersections of human labor to climate and environment. Farmers in the region spend much of their time hoping for more rain while fishermen often want less rain and the right kinds of wind. Even though there is fluid movement among the three subsistence modes, there are still farming, foraging, and fishing modes associated with Masikoro, Mikea, and Vezo identities, so these communities are likely to maintain somewhat different knowledge.

The results of Study 1 also suggest that there may be interesting differences in men and women’s causal knowledge. As stated above, we do not have a good explanation for why male focus groups exhibited greater agreement in risk ratings than did female focus groups, especially considering that men and women grow the same crops and forage for most of the same terrestrial resources. Perhaps there are differences in women’s mobility and knowledge of activities in distant subsistence modes. It is also possible that men and women communicate ecological knowledge in different ways.

Our data do not address whether southwestern Malagasy apply integrative thinking beyond the domain of subsistence, where the hypervariable climate is not a direct influence on outcomes. In a domain not so obviously connected to climate, that of death and afterlife, Astuti and Harris (2008) found that Vezo adults tended to explain death as biological cessation in the context of a story about a malaria death in a hospital while explaining death as continuity into the afterlife of ancestors in the context of a story about a funeral. Astuti and Harris (2008), like us, argue that biology and cosmology are not competing causal explanations, but they do not present evidence whether or how these causal models are integrated. Death in Madagascar is a risky venture, as the dead cannot control how or where their body will be handled or buried. It would be interesting to learn whether risk and choice in this domain mirror or intersect with subsistence decisions.

The pattern of integrative thinking that we have documented for southwestern Madagascar echoes Evans-Pritchard’s (1937) classic description of Zande witchcraft as an idiom of causality. Is a hypervariable environment the reason for Zande integrative thinking? The closest weather station to Zandeland in Dewar and Richard’s (2007) comparative climate study was Yei, South Sudan, which has a monthly rainfall predictability score of 0.527, nearly double that of Toliara, Madagascar 0.281. It is unclear how much unpredictability, and unpredictability of what, exactly, we would expect to be associated with integrative thinking. Evans-Pritchard argued that Zande witchcraft is a domain-general causal model, applied not only to subsistence, but health, politics, and domestic life. “There is no niche or corner of Azande culture that [witchcraft] does not twist itself” (Evans-Pritchard, 1937, p. 63). One possibility is that Zande witchcraft helps people deal with a more domain-general form of unpredictability, perhaps relating to social alliances. It may also be that integrative thinking can be a tool to solve more than one set of ecological challenges.

The causal flow depicted in **Figure 2** is not dissimilar from what one might expect in Christianity and other Abrahamic cosmologies. Just as a southwestern Malagasy person must beg ancestors and other spirits to beg God for good fortune, so a Catholic calls upon Jesus, the Virgin Mary, and Saints to intercede on her behalf. We suspect that when southwestern Malagasy convert to Christianity they simply insert Jesus into the mediating role alongside ancestors and other spirits. Indeed, it is not uncommon to hear our informants refer to Jesus as the *razambazaha*, “the foreigners’ (or white people’s) ancestor.” Thus “conversion” may result in minimal changes to cosmology, culture, and behavior, which is consistent with our observations that even clan heads, diviners, and spirit mediums are sometimes ardent churchgoers.

Our interpretation that for southwestern Malagasy “natural” and “supernatural” are distinct categories of causes and yet integrated into a single causal model requires further discussion and future research, given contradictory statements in the literature that natural and supernatural knowledges are either inherently different or that the natural/supernatural dichotomy is a figment of Western imagination. Boyer (2000) has argued that causal knowledge of the natural world is an extension of innate intuition while supernatural causality, by definition, involves counter-intuitive twists on natural relationships that are learned later in life. By contrast, some cultural anthropologists have warned that dichotomies like mind/body, nature/culture, and natural/supernatural are cultural artifacts of European Enlightenment philosophy that are not shared by many non-Western traditional cultures (Ingold, 1991, p. 362; Ortner, 1996; Lambek, 1998). Some anthropologists of religion argue against the utility of the natural/supernatural dichotomy, stating that many peoples see no difference in the realness of rocks and rain versus ghosts and angels, and may see all such forces as similarly animate and agentive (Lambek, 2008, pp. 5–7).

Whether non-Western peoples dichotomize natural/supernatural or other such knowledges is ultimately an empirical question. The data presented here show that different kinds of questions yield different sets of causes, suggesting that they are distinct, but they are not dichotomous or opposed categories because they do not contradict each other. Our data do not address the question of what makes a causal force like rainfall or ancestors fit into one category or the other, nor whether “natural” and “supernatural” mean the same thing to our informants as these terms generally indicate in Western discourse.

A seemingly obvious difference between natural and supernatural is that the supernatural is the domain of invisible agents, where agents, after Leslie (1995), have mechanical properties of force and energy, goal orientation, and cognition. Natural causes, by contrast, are visible agents (pests), invisible non-agents (wind), or visible non-agents (rainfall). However, it is not difficult to conjure contradictory examples. The Malagasy astrological calendar is supernatural in that it provides what Howell (2012) calls “cosmo-rules” to honor ancestors, possessing spirits, and God, but astrology is not agentive for it does not think or pursue goals. The germ theory of disease constitutes a natural force but involves invisible agents.

Could it be that when southwestern Malagasy discuss climatic causes within their natural submodel or level of causality, they understand “rainfall” and “wind” to be personified agents with goal orientation and cognition, similar to how they think about ancestors and God? When the first author suggested this “animistic” possibility to the Malagasy co-authors, they initially found the question difficult to understand. We also found this to be a difficult question to pose to Vezo informants. Wind and rainfall, they insisted, were just natural. But to the first author’s confusion, the word “natural” in Malagasy is *voajanahare*, which translates literally as, “fruit of God” (work of God). Further research is required to understand exactly what separates forces like rain, wind, pests, and germs from ancestors, spirits, God, and astrology.

## Conclusion

This investigation of coexistence thinking was framed around an apparent paradox in the ecology and culture of subsistence risk in rural southwestern Madagascar. Crops fail and prey become scarce because of climatic factors, which are highly unpredictable; Masikoro, Mikea, and Vezo adapt to their hypervariable environment via diversification and behavioral flexibility. And crops fail and prey become scarce because of the judgments and moods of spiritual agents; southwestern Malagasy adapt to their high spiritual insecurity by narrowing their range of behaviors, by following a pious code of taboos, ritual prescriptions, and astrological proscriptions. One possible cognitive explanation for this paradox is that southwestern Malagasy maintain contradictory natural and supernatural causal models in their heads and employ each in certain circumstances. A second possibility is that they see no paradox, for wind, rain, God, and ancestors belong to the same category of causes that permits behavioral flexibility and pious conformity in different circumstances. We interpret the results of the three studies presented here as supporting a third, integrative model, where ecological factors explain why crops and fields fail, supernatural factors explain why persons succeed or fail, and supernatural factors influence economic outcomes via natural factors. We have argued that this permits Masikoro farmers, Mikea foragers, and Vezo fishers to effectively share ecological information undistorted by personal concerns, while simultaneously giving reason for why ecological knowledge fails without casting doubt about the validity of the ecological knowledge. This is of course a functionalist explanation; our data do not address the origins of this purported cognitive adaptation to a hypervariable environment.

It is difficult to disentangle the influences of ecology and culture on coexistence thinking with a single synchronic case study. Culturally learned causal rules are part of how people adapt to environmental challenges (Steward, 1955; Richerson and Boyd, 2005), and it could be that integrative thinking is a mental strategy developed rather recently by the ancestors of today’s Masikoro, Mikea, and Vezo, or perhaps a broader geographical range of Malagasy people. Yet cultural traditions may persist that have neutral or negative effects on people’s survival (Durham, 1991); it is possible that southwestern Malagasy integrative thinking is not adaptive, or did not develop here because of its adaptive value, but was simply inherited from previous generations as part of broader Austronesian and East-African culture histories. This echoes a classic debate in cultural ecology as to whether a society’s religious ideas are peripheral with regards to ecological adaptation (Steward, 1955) or central to it (Rappaport, 1968). Future research could explore people’s application of coexistence thinking to subsistence risk through cross-cultural comparison, by contrasting samples from places with different levels of climate unpredictability, both within Madagascar where people share similar traditional beliefs, within the broader Austronesian tradition of which Madagascar is a part, and in distantly or unrelated cultures.

Researchers studying choice under risk within the economic tradition have traditionally applied probability theory and rational actor models to decision-analyses, implicitly promoting a psychic unity of humankind by which all peoples make similar decisions for similar reasons. Our research suggests that risk-sensitive decision-making may depend on the local ecology and culture and whether people employ target-dependent, synthetic, or integrative thinking. This argument emphasizes cultural relativism while simultaneously providing a framework for the kinds of cultural-cognitive variations we may expect to find in different settings.

## Conflict of Interest Statement

The authors declare that the research was conducted in the absence of any commercial or financial relationships that could be construed as a potential conflict of interest.

## Footnotes

1 Interpersonal magical attacks (sorcery) compose a parallel moral system. Returning to the example of the man who discovers though divination that his jealous neighbor has killed his livestock, the man could ask the diviner to encourage the spirits to cause harm to the wrongdoing neighbor. Only a minority of sorcerers are capable of attack magic (*vorike*), for it requires “dirty” magical substances (*volohazomaloto*) and is generally considered to be immoral. The moral code enforced by sorcery may encourage niceness (to avoid attacks by neighbors) as Evans-Pritchard (1937) argued for Zande witchcraft; but, judging by the amount of gossip we hear on the topic and the ubiquity of the protective magic that people wear, sorcery seems to encourage suspicion, envy, enmity, and discord.

2 In the risk definitions, seven responses used the term *risike*, including at least one Masikoro, one Mikea, and one Vezo speaker. Two responses used *kitahitahy*, both Mikea (although two other Mikea used *risike*). One speaker, a Masikoro woman, used both terms.

3 Reolo means “Mr. Person” and Tsiato means “not here.” These equivalents of “John Doe” are nonetheless realistic names.

4 Bevondrorano was a well-known subsistence environment for these Mikea families even before they were displaced by the Park, as many lived part of the year here to sharecrop manioc, do rice wage labor, dig wild *babo* tubers (*Dioscorea acuminata*) and sell game to savanna people. Bevondrorano people have many Mikea kin among the greater Bevondro community. They were not displaced by force, but by policies by Madagascar National Parks that made forest life more complicated.

5 The wealth score included two assets that are most commonly owned by Masikoro and Mikea (land, cattle) and two commonly owned by Vezo (ocean-going canoes, shark fishing nets). The other assets were equally likely to be owned by anyone with financial capital: oxcart, radio, generator, and telephone.

6 The procedure we eventually developed was, after asking about causal influences of pairs of cards, to graduate to combinations of three cards, then finally, the fourth.
