# Supplementary material for: Ecological and cosmological coexistence thinking in a hypervariable environment: causal models of economic success and failure among farmers, foragers, and fishermen of southwestern Madagascar
Source: Front Psychol. 2015 Oct 13;6:1533. doi: 10.3389/fpsyg.2015.01533 (PMC4602100; doi:10.3389/fpsyg.2015.01533)
Supplement: Supplementary file 1 [file Table_1.DOCX]

**Ecological and cosmological coexistence thinking in a hypervariable environment: Causal models of economic success and failure among farmers, foragers, and fishermen of southwestern Madagascar**

Bram Tucker*^1^, Tsiazonera (no first name)^2^, Jaovola Tombo^3^, Patricia Hajasoa^4^, Charlotte Nagnisaha^5^

**Supplemental material**

Provided here is the text of the scripts used in vignettes in study 2, the southwestern dialect of Malagasy and an English translation.

| **Economic version, Mikea**  "Intony misy tantara mikasike olo roe, Reolo roze amin'ny Tsiato. Reolo noho Tsiato olo mpinama mare, fa tsy mpilongo marinike sady samby mana gny tana misy azy. Samby olo mihetsiketsike no sady mamelon-po roze roe. Matetike roze mifankahita antsena egny, miarake mino kafe le miresadresake sy mifanontane vaovao.  "Ndraike andro, niresake momba ty fiainan-droze roze roe, bakeo hay fa Reolo nahavagno mare tany re Tsiato ndra tsako, ndra vary, ndra balahazo, vokatse iaby. Tsiato nefa tsy nahavokatse firy." | **Economic version, Mikea**  "This is the story of two people, Reolo and Tsiato. Reolo and Tsiato are good friends, but they are not close relatives, and they live in distant villages. But both of them work hard to support their livelihood. They often see each other in the marketplace, where they drink coffee and exchange news.  "One day, exchanging information about themselves, they realize that Reolo harvests a lot more than Tsiato, whether maize, rice, manioc; all crops. Tsiato does not harvest much at all." |
| --- | --- |
| **Economic version, Vezo**  "Intony misy tantara mikasike olo roe, Reolo roze amin'ny Tsiato. Reolo noho Tsiato olo mpinama mare, fa tsy mpilongo marinike sady samby mana gny tana misy azy. Samby olo mihetsiketsike no sady mamelon-po roze roe. Matetike roze mifankahita antsena egny, miarake mino kafe le miresadresake sy mifanontane vaovao.  "Ndraike andro, niresake momba ty fiainan-droze roze roe, bakeo hay fa Reolo nahazo tindroke mare tany re Tsiato tsy ho fia zay, tsy ho horita, tsy ho janga, raha iaby. Tsiato nefa tsy nahazo firy." | **Economic version, Vezo**  "This is the story of two people, Reolo and Tsiato. Reolo and Tsiato are good friends, but they are not close relatives, and they live in distant villages. But both of them work hard to support their livelihood. They often see each other in the marketplace, where they drink coffee and exchange news.  "One day, exchanging information about themselves, they realize that Reolo harvests a lot more than Tsiato, whether fish, octopus, sea cucumbers; everything. Tsiato does not harvest much at all." |
| **Religious version, Mikea**  "Intony misy tantara mikasike olo roe, Reolo roze amin'ny Tsiato. Reolo noho Tsiato olo mpinama mare, fa tsy mpilongo marinike sady samby mana gny tana misy azy. Samby mamonjy raharaham-pianakavia ndra savatse, ndra soronanake, ndra faty... ary mififankahita matetike roze an-kavoria ao ka miresadresake sy mifanontane vaovao.  "Ndraike andro, niresake momba ty fiainan-droze roze roe, bakeo hay fa Reolo nahavagno mare tany re Tsiato ndra tsako, ndra vary, ndra balahazo, vokatse iaby. Tsiato nefa tsy nahavokatse firy." | **Religious version, Mikea**  "This is the story of two people, Reolo and Tsiato. Reolo and Tsiato are good friends, but they are not close relatives, and they live in distant villages. But both of them attend a lot of family ceremonies, such as circumcision, rites of filiation, and funerals.... they often see each other in the ceremonies, where they exchange news.  "One day, exchanging information about themselves, they realize that Reolo harvests a lot more than Tsiato, whether maize, rice, manioc; all crops. Tsiato does not harvest much at all." |
| **Religious Version, Vezo**  "Intony misy tantara mikasike olo roe, Reolo roze amin'ny Tsiato. Reolo noho Tsiato olo mpinama mare, fa tsy mpilongo marinike sady samby mana gny tana misy azy. Samby mamonjy raharaham-pianakavia ndra soro, ndra soronanke, ndra faty.. ary mififankahita matetike roze an-kavoria ao ka miresadresake sy mifanontane vaovao.  "Ndraike andro, niresake momba ty fiainan-droze roze roe, bakeo hay fa Reolo nahazo tindroke mare tany re Tsiato tsy ho fia zay, tsy ho horita, tsy ho janga, raha iaby. Tsiato nefa tsy nahazo firy." | **Religious Version, Vezo**  "This is the story of two people, Reolo and Tsiato. Reolo and Tsiato are good friends, but they are not close relatives, and they live in distant villages. But both of them attend a lot of family ceremonies, such as invocation of ancestors, rites of filiation, and funerals.... they often see each other in the ceremonies, where they exchange news.  "One day, exchanging information about themselves, they realize that Reolo harvests a lot more than Tsiato, whether fish, octopus, sea cucumbers; everything. Tsiato does not harvest much at all." |
